# Effects of Wheat Matrix and Jujube Incorporation Form on the Quality and Antioxidant Properties of Whole-Wheat Steamed Bread

**DOI:** 10.3390/foods15213769

**Published:** 2026-10-22

**Authors:** Ruijing He, Jingyi Zhang, Mengqin Li, Shunshun Lin, Daowen Wang, Kunpu Zhang, Yan Ma, Shanshan Qu, Xiaoling Tian

**Affiliations:** 1 College of Food Science and Technology, Henan Agricultural University, Zhengzhou 450002, China; 2 National Wheat Engineering Research Center, College of Agronomy, Henan Agricultural University, Zhengzhou 450002, China; 3 Postdoctoral Station of Crop Science, College of Food Science and Technology, Henan Agricultural University, Zhengzhou 450002, China

**Keywords:** anthocyanins, colored wheat, jujube, texture profile analysis, whole-wheat steamed bread

## Abstract

The incorporation form of fruit-derived ingredients may influence the quality and antioxidant responses of whole-wheat steamed bread. Jujube was incorporated into white- and colored-wheat whole-wheat flours either as dry powder or as prehydrated pulp at 5%, 10%, and 15% on a dry-matter basis, while the total water addition was kept constant across all formulations. Product geometry, surface color, texture, cross-sectional appearance, anthocyanin content, and ABTS radical scavenging activity were evaluated. Specific volume showed relatively limited variation across the tested formulations, including a range of 1.59–1.67 mL/g in the white-wheat powder series. Colored-wheat flour contained 4.24 mg/100 g anthocyanins compared with 0.99 mg/100 g in white-wheat flour, and anthocyanin content in steamed bread was governed mainly by wheat matrix. ABTS activity was affected by wheat matrix, jujube form, addition level, and their two-way interactions, reaching 66.15% in colored-wheat steamed bread containing 15% jujube pulp. Among the formulations tested, 10% jujube pulp in the colored-wheat system provided a more balanced response in product shape and antioxidant activity, whereas increasing the level to 15% further enhanced ABTS activity but reduced several instrumental texture attributes.

## 1. Introduction

Whole-wheat products retain bran- and germ-derived dietary fiber, minerals, and phytochemicals that are largely removed during refined flour production [1,2,3]. Their use in steamed bread, however, also presents technological challenges. Bran competes with gluten and starch for water and physically interferes with gluten continuity, which can affect dough development and lead to lower volume, firmer texture, and a less uniform crumb structure [1,2,3]. Recent studies further show that the response of whole-wheat steamed bread to added ingredients is often dependent on ingredient type and addition level rather than following a simple linear trend [4,5,6,7,8].

Fruit-derived ingredients introduce both dietary fiber and polyphenols into this already complex matrix. Dietary fiber can redistribute water and physically interfere with gluten development, whereas polyphenols may interact with gluten proteins mainly through hydrogen bonding and hydrophobic interactions and, under oxidative conditions, may also participate in covalent reactions [4,9,10]. During fermentation and proofing, these effects occur alongside continued gluten development and water redistribution; during steaming, thermal treatment fixes the protein–starch matrix and may alter the stability of heat-sensitive phenolic compounds [9,10,11]. These processes provide a basis for understanding why the effects of fruit-derived ingredients may vary with their composition, physical state, and addition level. Colored wheat differs from conventional white wheat primarily in its phytochemical profile. Pigmented wheat varieties contain substantially higher levels of anthocyanins and other phenolic compounds, which contribute to their antioxidant-related properties [12,13]. These compounds are sensitive to processing conditions, and their retention during steamed-bread processing may also be influenced by interactions with proteins and other components of the wheat matrix [11,12,14,15]. Thus, colored whole-wheat flour represents a compositionally distinct processing matrix rather than simply a differently colored counterpart of conventional whole wheat [15,16].

Jujube (*Ziziphus jujuba* Mill.) contains sugars, dietary fiber, minerals, phenolic compounds, and other antioxidant-active constituents [17,18,19]. Its incorporation into wheat-based products may consequently affect both product quality and antioxidant-related properties. Recent work on jujube-enriched steamed bread has also shown addition-level-dependent changes in texture and antioxidant activity [20]. However, most previous studies have examined a single jujube form or a single wheat matrix, and little information is available on whether the same jujube material produces comparable responses when incorporated as dry powder or prehydrated pulp into white- and colored-wheat whole-wheat systems.

Accordingly, the present study investigated the individual and combined effects of wheat type, jujube incorporation form, and addition level on whole-wheat steamed bread. Product geometry, surface color, instrumental texture, crumb appearance, anthocyanin content, and ABTS radical scavenging activity were evaluated. The study was designed to determine whether the response to jujube incorporation form depends on wheat matrix and addition level and to clarify the resulting trade-offs between physical quality and antioxidant performance.

## 2. Materials and Methods

### 2.1. Materials

Colored wheat (Yuzhou Black Wheat No. 2) and white wheat (Jimai 22) were provided by the College of Agronomy, Henan Agricultural University (Zhengzhou, China). Commercial dried Xinjiang Huizao jujube (*Ziziphus jujuba* Mill. cv. Huizao) was purchased from a local market in Zhengzhou, China. The same batch of dried jujube was used for both powder and pulp preparation. According to the compositional information of the raw material used in this study, dried jujube contained 14.71% starch, 2.79% protein, 0.60% fat, and 6.39 mg/100 g anthocyanins. Active dry yeast was supplied by Yeast Co., Ltd. (Yichang, China).

### 2.2. Preparation of Whole Wheat Flour and Jujube Ingredients

Whole-wheat flour was prepared by grinding wheat kernels in a hammer cyclone mill until all material passed through an 80-mesh sieve (≤180 μm). Jujube powder was prepared by grinding dried jujube and passing it through a 60-mesh sieve. For the pulp treatments, the amount of dried jujube corresponding to each target addition level was blended with a portion of the formulation water using a Joyoung blender (JYL YZ01, 1000 W Joyoung Co., Ltd., Jinan, Shandong, China) under the preset blending program for approximately 2–3 min until a homogeneous pulp with no obvious coarse particles was obtained. The water used for pulp preparation was included in the total formulation water.

### 2.3. Preparation of Jujube-Enriched Whole Wheat Steamed Bread

Steamed bread was prepared with minor modifications according to recent studies on enriched and whole-wheat Chinese steamed bread [1,4]. Jujube was incorporated into colored-wheat and white-wheat whole-wheat flours at 5%, 10%, and 15% on a on a flour-weight basis, either as dry powder or as prehydrated pulp. Yeast was added at 1% (flour basis). The total added water was fixed at 52% of flour weight for all formulations. For powder treatments, the required water was added during dough mixing. For pulp treatments, part of the same total water was first used to prepare the jujube pulp, and only the remaining water was subsequently added during dough mixing. For pulp preparation, the amount of water allocated to blending was adjusted as needed to obtain a homogeneous and readily blendable pulp, rather than being fixed at a predetermined jujube-to-water ratio. Therefore, the sum of the water used for pulp preparation and the water added during dough mixing was identical to the total water used in the corresponding powder treatments.

The dough was mixed for 10 min, fermented at 35 °C and 85% relative humidity for 1 h, divided into 50 g pieces, and passed through the sheeting step eight times to obtain a uniform dough sheet; the same number of passes was used for all formulations. The dough pieces were then manually molded, proofed at 35 °C and 85% relative humidity for 30 min, and steamed in a covered steamer over boiling water for 15 min. After steaming, the samples were cooled to room temperature before analysis.

### 2.4. Determination of Proximate Composition and Wet Gluten of Whole Wheat Flour

Starch content was determined according to AACC 76-13.01. Protein content was determined according to AACC 46-12.01. Total dietary fiber was determined according to AACC 32-05.01. Crude fat was determined according to AACC 30-25.01. Ash content was determined according to AACC 08-01.01. Wet gluten content was determined using the mechanical washing procedure described in AACC 38-12.02.

### 2.5. Determination of Fe, Zn, and Se in Whole Wheat Flour

Mineral elements (Fe, Zn, and Se) in whole-wheat flours were determined according to GB 5009.268-2016 using inductively coupled plasma mass spectrometry (ICP-MS). Approximately 0.5 g of ground sample was mixed with 5 mL of nitric acid and subjected to microwave digestion. After digestion, the solution was diluted to 25 mL with ultrapure water. Element concentrations were determined using an ICP-MS instrument (Agilent Technologies, Santa Clara, CA, USA). Quantification was performed using external calibration with internal-standard correction, and reagent blanks were analyzed in parallel. Results were expressed as μg/g sample.

### 2.6. Determination of Total Anthocyanin Content

Total anthocyanin content was determined using a modified acidified-methanol extraction method [21]. Flour samples were analyzed directly, whereas steamed-bread samples were freeze-dried and ground into powder before extraction. Approximately 0.5 g of sample was mixed with extraction solvent at a solid-to-solvent ratio of 1:10 (*w*/*v*). The extraction solvent consisted of methanol and 1 mol/L HCl (85:15, *v*/*v*). The mixture was extracted in the dark for 30 min and centrifuged at 8000 rpm. The residue was re-extracted with acidified methanol at a ratio of 1:5 (*w*/*v*), and the supernatants were combined. Absorbance was measured at 535 nm using acidified methanol as the blank. Quantification was performed using an external calibration curve of cyanidin-3-*O*-glucoside. Results were expressed as mg cyanidin-3-*O*-glucoside equivalents per 100 g sample. For steamed-bread samples, results were calculated on the basis of the freeze-dried sample; therefore, no additional moisture correction was applied.

### 2.7. Determination of ABTS Radical Scavenging Activity

The freeze-dried steamed-bread powder described in Section 2.6 was used for the ABTS assay. ABTS radical scavenging activity was determined following the method of Re et al. [22] with minor modifications. Briefly, 7 mmol/L ABTS solution was mixed with 2.45 mmol/L potassium persulfate solution and kept in the dark at room temperature for 12–16 h to generate the ABTS radical cation. Before use, the working solution was diluted to an absorbance of 0.70 ± 0.05 at 734 nm. Then, 0.4 mL of sample extract was mixed with 3.0 mL of diluted ABTS working solution. After standing for 30 min, absorbance was measured at 734 nm. Methanol (0.4 mL) mixed with 3.0 mL diluted ABTS working solution was used as the control. ABTS radical scavenging activity was calculated as follows Equation (1). ABTS•+ scavenging activity was expressed as percentage scavenging activity; therefore, no additional moisture correction was calculated using Equation (1):

(1) $$\mathrm{ABTS}•+\;\mathrm{scavenging}\;\mathrm{activity}\;(\%)\;=\;\frac{A_{0}-A_{1}}{A_{0}}\times 100$$

where A_0_ is the absorbance of the control containing 0.4 mL methanol and 3.0 mL ABTS•+ working solution, and A_1_ is the absorbance of the sample solution.

### 2.8. Specific Volume and Height-to-Diameter Ratio of Steamed Bread

Specific volume was determined by a seed displacement method adapted from AACC 10-05.01. Each steamed bread sample was weighed and its volume was measured by displacement using millet as the filling medium. The same millet and filling procedure were used for all samples. Specific volume was expressed as mL/g.

The height and diameter of each steamed bread were measured with a vernier caliper, and the height-to-diameter ratio was calculated using Equation (2):

(2) $$\mathrm{Height}\text{-}\mathrm{to}\text{-}\mathrm{diameter}\;\mathrm{ratio}\;=\;\frac{H}{D}$$

where H is the height of the steamed bread and D is its maximum diameter measured at the widest part.

### 2.9. Color Measurement

Surface color of the steamed bread samples was measured using an NR110 colorimeter (Shanghai Jiance Industry Co., Ltd., Shanghai, China), and the results were recorded as L* (lightness), a* (redness), and b* (yellowness). Measurements were performed on the upper surface of the steamed bread as described in recent studies on steamed-bread quality evaluation [4].

### 2.10. Texture Profile Analysis

Texture profile analysis (TPA) was carried out on slices (20 mm thickness) taken from the central portion of the steamed bread using a texture analyzer equipped with a P/50 probe, following recent steamed-bread quality studies [5]. The test conditions were as follows: pre-test speed, 1.0 mm/s; test speed, 2.0 mm/s; post-test speed, 1.0 mm/s; compression ratio, 50%; and trigger force, 5 N. For each formulation, dough units were prepared separately and steamed under the same standardized conditions. TPA was performed on central slices of the resulting steamed-bread samples, with six measurements obtained for each formulation and the mean value used for statistical analysis.

### 2.11. Crumb Cross-Sectional Structure

After steaming and cooling to room temperature, the samples were kept sealed until imaging. The central portion of each steamed bread was cut and directly scanned using a flatbed scanner. The resulting cross-sectional images were used as representative visual documentation of the general crumb appearance.

### 2.12. Statistical Analysis

The experiment followed a 2 × 2 × 3 factorial design with wheat type (white wheat and colored wheat), jujube form (powder and pulp), and addition level (5%, 10%, and 15%) as the three factors. Results are presented as mean ± standard deviation. Three-way ANOVA was performed to evaluate the main effects of wheat type, jujube form, and addition level and their two-way and three-way interactions. Treatment-level comparisons presented in individual figures and tables were analyzed separately using one-way ANOVA followed by Duncan’s multiple range test, as specified in the corresponding figure captions or table notes. Statistical significance was set at *p* < 0.05. PCA and Pearson correlation analyses were performed using the 12 treatment means derived from the 2 × 2 × 3 factorial design after variable standardization. Three-way ANOVA and one-way ANOVA were performed using SPSS 22.0, while PCA, Pearson correlation analysis, and figure preparation were performed using Origin 2024.

## 3. Results and Discussion

### 3.1. Flour Matrix Characteristics

The two whole-wheat flours showed comparable starch contents, but differed markedly in several compositional traits (Table 1 and Table 2). Compared with the white-wheat flour, the colored-wheat flour contained higher protein, fat, ash, and wet gluten contents, whereas the white-wheat flour showed a higher dietary fiber content. These differences indicate that the two flours should be regarded as distinct processing matrices rather than simply two color variants of the same raw material. The higher wet gluten content of the colored-wheat flour was consistent with its higher protein content and may contribute to the subsequent differences in product geometry and texture [1,2,5]. Similar matrix-dependent differences have been emphasized in recent studies on whole-wheat steamed bread, bran-modified dough systems, and colored wheat products [2,5].

The two flours also showed distinct mineral and phytochemical profiles. White-wheat flour contained higher Fe and Zn contents, whereas colored-wheat flour contained higher Se and anthocyanin contents. The anthocyanin content of the colored-wheat flour was more than four times that of the white-wheat flour, providing a stronger antioxidant-related background for the subsequent steamed-bread formulations. This observation is consistent with recent reports showing that colored wheat can deliver a markedly higher phytochemical potential than common wheat, especially when anthocyanin-rich genotypes are used [12,13,23]. In the present work, these initial flour differences provided the compositional basis for the later divergence in color, texture, and antioxidant-related responses of steamed bread.

### 3.2. Specific Volume and Height-to-Diameter Ratio

**Figure 1:**
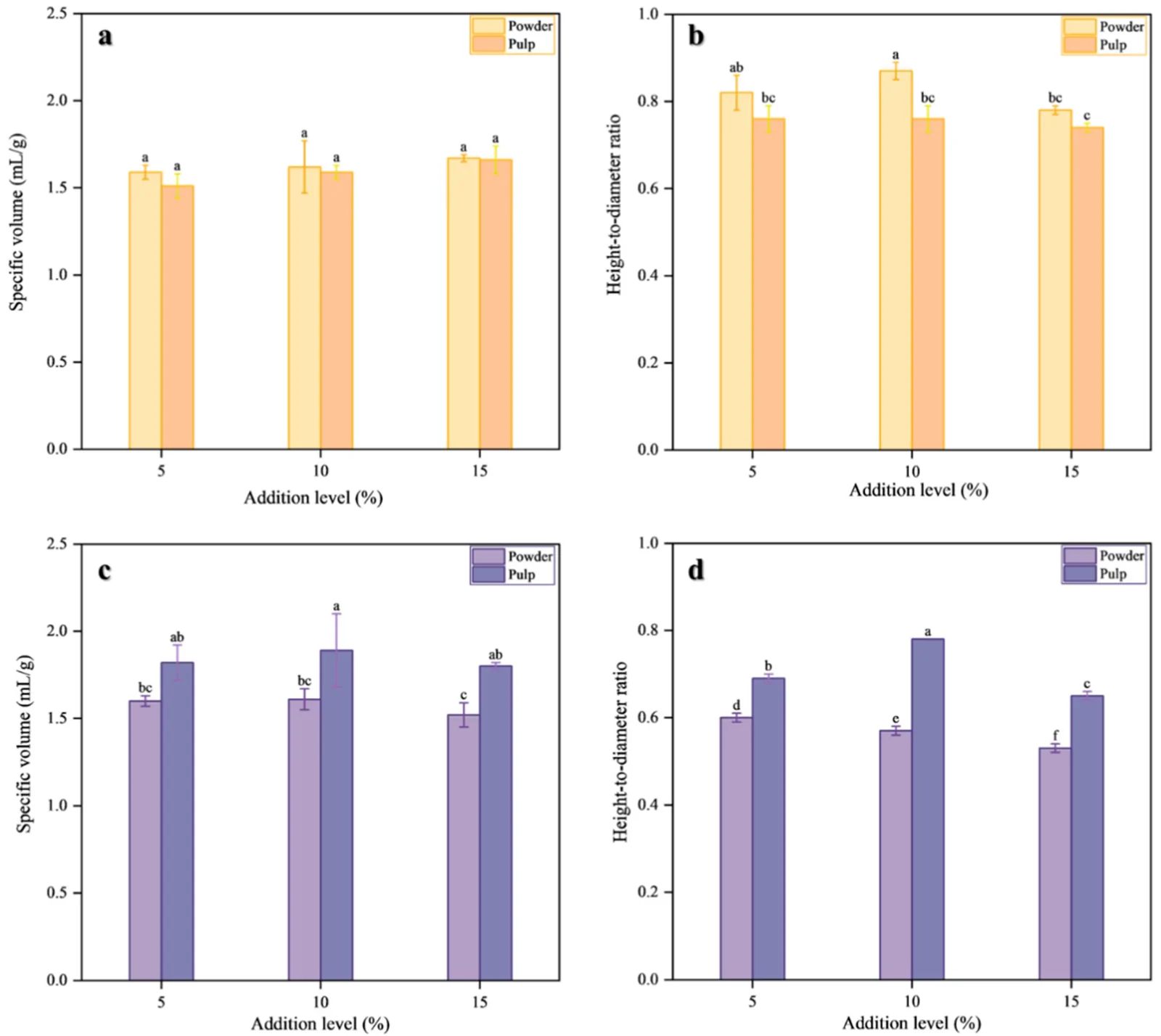
Effects of jujube addition form and level on (**a**,**c**) specific volume and (**b**,**d**) height-to-diameter ratio, of whole-wheat steamed bread prepared from (**a**,**b**) white wheat and (**c**,**d**) colored wheat. Values are presented as mean ± SD (*n* = 3). Different lowercase letters indicate significant differences among the six treatment combinations within the same panel (*p* < 0.05).

As shown in Figure 1, within the tested 5–15% addition range, specific volume varied within a relatively narrow range, whereas the height-to-diameter ratio was more sensitive to treatment. In the white-wheat powder series, specific volume remained within a narrow range (1.59–1.67 mL/g), with no marked reduction as the addition level increased. A similar pattern was observed in the white-wheat pulp series, although the 15% pulp treatment showed the highest mean specific volume. In the colored-wheat system, powder addition also caused only modest changes in specific volume, while the pulp series reached the highest mean value at 10% addition. These results indicate that, within the 5–15% addition range, increasing jujube level was not the dominant factor affecting specific volume. This agrees with several recent steamed-bread studies in which ingredient enrichment altered dough structure and texture more readily than bulk volume [4,6,24,25].

Compared with specific volume, the height-to-diameter ratio showed clearer formulation responses. In the white-wheat powder series, the ratio increased from 5% to 10% and then decreased at 15%, with the highest height-to-diameter ratio observed at the intermediate addition level. In the white-wheat pulp series, the ratio remained comparatively stable. In contrast, the colored-wheat powder series showed a progressive decline in height-to-diameter ratio with increasing addition level, while the colored-wheat pulp series exhibited a peak at 10% followed by a decrease at 15%. This non-linear response may reflect a balance between the dispersion of the prehydrated jujube material and increasing interference of jujube solids with the dough matrix at the higher addition level. Taken together, these results suggest that product shape was more sensitive than specific volume to the combined effects of wheat matrix and jujube form. From a practical standpoint, the colored-wheat pulp treatment showed the highest height-to-diameter ratio at 10% addition, whereas the ratio was lower at 15% addition.

Three-way ANOVA further showed that specific volume was significantly affected by wheat type, jujube form, and their interaction, but not by addition level (Table 3). The significant wheat type × jujube form interaction indicates that the effect of powder and pulp was not identical in the two wheat matrices. In contrast, interactions involving addition level were not significant for specific volume, which is consistent with its relatively narrow variation across the 5–15% range. The height-to-diameter ratio showed a stronger factorial response, with significant effects of wheat type, jujube form, addition level, wheat type × jujube form, and the three-way interaction. Thus, the influence of jujube incorporation form on product shape depended on both wheat matrix and addition level. Similar concentration-dependent responses have recently been reported for other functional ingredients incorporated into whole-wheat steamed bread [4,6,7,24,25].

### 3.3. Surface Color

**Figure 2:**
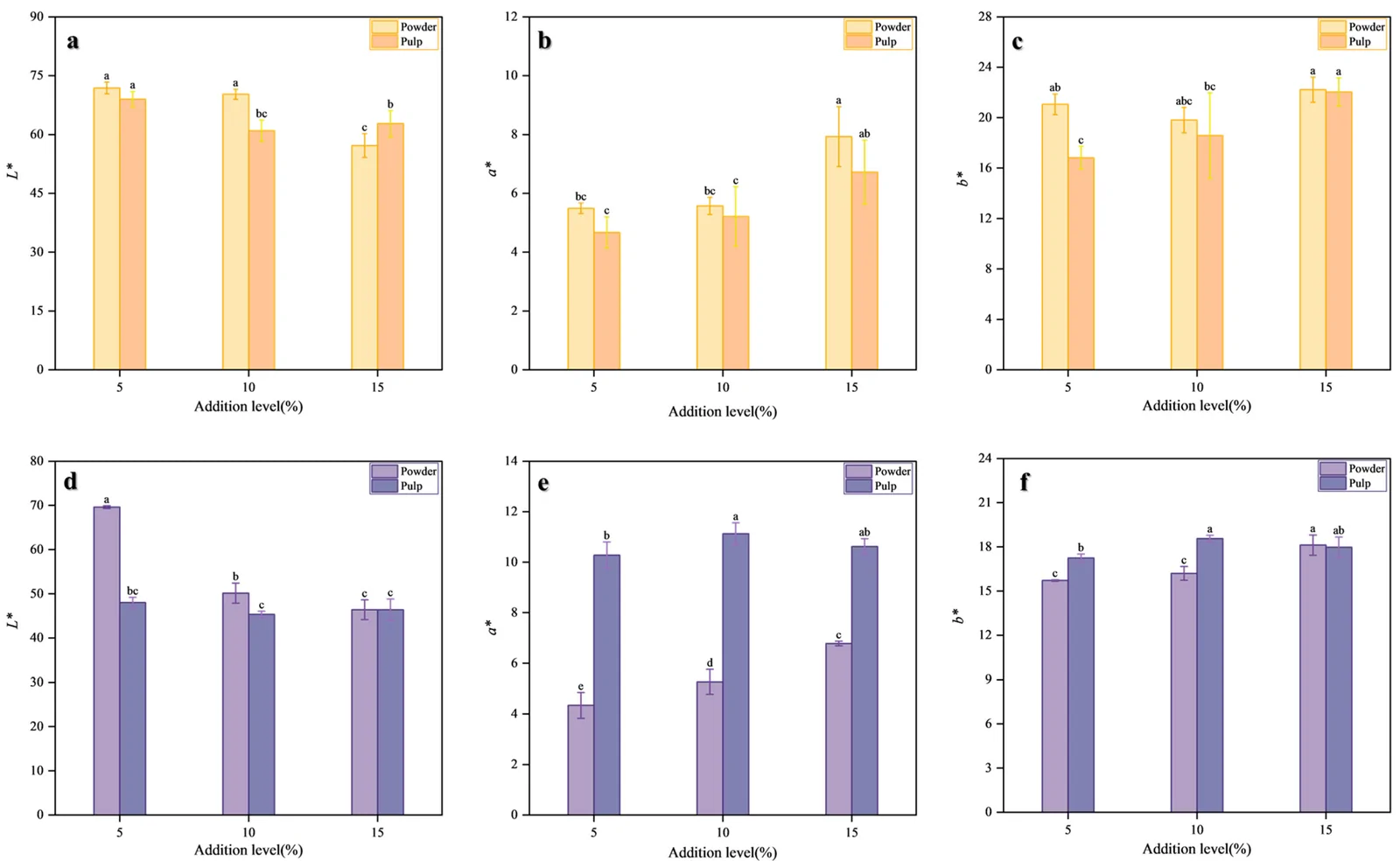
Effects of jujube addition form and level on the surface color parameters of whole-wheat steamed bread prepared from white and colored wheat: (**a**,**d**) L*, (**b**,**e**) a*, and (**c**,**f**) b*. Values are presented as mean ± SD (*n* = 3). Different lowercase letters indicate significant differences among the six treatment combinations within the same panel (*p* < 0.05).

Surface color changed much more noticeably than product geometry (Figure 2). In both wheat systems, increasing jujube addition generally reduced L* and increased a*, indicating darker products with a stronger red-brown tone. This trend was especially evident at the highest addition level and became more pronounced when jujube was incorporated as pulp. The stronger color response of the pulp treatments may be related to their prehydrated state. Blending dried jujube with a portion of the formulation water before dough mixing may facilitate the release and dispersion of pigments and other soluble components, resulting in a more uniform color contribution than dry powder. Because total formulation water was fixed, this difference cannot be attributed to a higher overall water addition in the pulp treatments.

In the white-wheat system, the powder-treated samples maintained relatively high L* values at 5% and 10%, but showed a sharp decrease at 15%, indicating that high powder addition markedly darkened the product surface. The white-wheat pulp series also showed a reduction in L*, although the shift was more gradual. Meanwhile, a* increased in both series, with the highest value appearing in the 15% white-wheat powder treatment. The b* response was more variable, but tended to increase at high jujube levels, especially in the pulp series. These results suggest that in the white-wheat matrix, jujube addition primarily acted as a color-intensifying factor, and the effect became stronger at higher inclusion levels.

The colored-wheat system displayed a much darker baseline. In the powder series, L* declined substantially from 69.61 at 5% to 46.39 at 15%, while a* rose from 4.34 to 6.72. In the pulp series, L* values remained low across all three addition levels, and a* values were consistently high, around 10–11, indicating a stable and intense reddish-brown appearance [12,13,15,16]. In contrast, b* changed less strongly in the colored-wheat samples. Overall, the colored-wheat + jujube-pulp combinations produced the most distinct visual profile. These combinations therefore produced the largest visual departure from conventional steamed bread. Whether these color differences influence consumer acceptance requires further sensory evaluation [26]. Comparable color shifts have been reported in steamed breads fortified with fruit powders, bran-rich materials, and polyphenol-rich ingredients [4,11,26].

The factorial analysis further confirmed that the color response depended on the combination of formulation factors rather than on individual factors alone. L* was significantly affected by all three main factors and all interaction terms, whereas a* was affected by the three main factors and all two-way interactions but not by the three-way interaction. For b*, significant wheat type × jujube form and three-way interactions were observed. These results are consistent with the visibly different color responses of powder and pulp in the two wheat matrices and indicate that the effect of increasing jujube level cannot be interpreted independently of wheat type and incorporation form. Similar matrix- and ingredient-dependent color changes have been reported in colored-wheat and fruit-enriched steamed bread systems [15,16].

### 3.4. Texture Profile Analysis and Crumb Structure

Texture results (Table 4) showed that the effects of jujube depended strongly on both wheat matrix and addition form. In the white-wheat powder series, hardness decreased progressively from 1494.25 g at 5% to 950.98 g at 15%, while springiness and resilience reached their highest values at 10%. These results indicate that the intermediate powder addition level was associated with higher springiness and resilience, whereas the 15% treatment showed lower hardness. In the colored-wheat powder series, hardness remained higher than in the corresponding white-wheat samples, and springiness, cohesiveness, and resilience were generally lower, indicating a firmer and less elastic structure. Similar texture responses have been reported in whole-wheat and fiber-enriched steamed bread, where water competition and disruption of gluten–starch continuity have been proposed as contributing factors [4,9,27,28,29].

A different response pattern was observed in the pulp-treated samples. In the white-wheat pulp series, hardness, gumminess, and chewiness were highest at 10%, whereas the 15% treatment exhibited the highest springiness and resilience. In contrast, hardness in the colored-wheat pulp series decreased from 2259.20 g at 5% to 1713.80 g at 15%, while springiness, cohesiveness, and resilience also decreased with increasing pulp level. The progressive decrease in hardness may reflect increasing interference of the prehydrated jujube material with the gluten–starch matrix as the pulp level increased. These contrasting trends provide a direct example of the significant interaction effects identified by the three-way ANOVA: increasing jujube pulp did not produce the same textural response in the two wheat matrices. This matrix-dependent behavior is consistent with recent reports showing that the effect of functional ingredients on steamed-bread texture depends strongly on ingredient level and the surrounding flour system [4,6,24,27,28,29].

**Figure 3:**
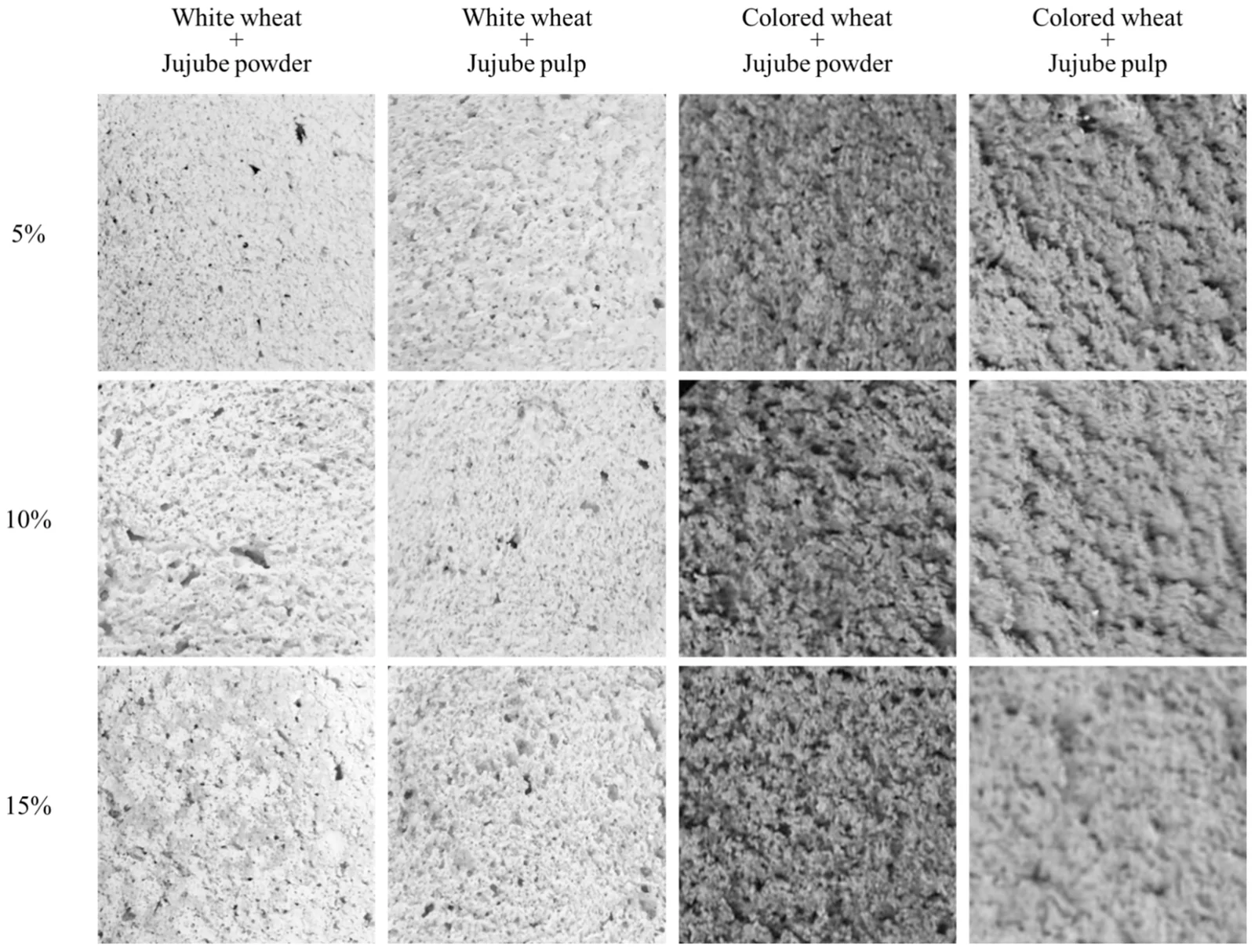
Representative crumb cross-sectional images of whole-wheat steamed bread prepared from white and colored wheat with jujube powder or jujube pulp at 5%, 10%, and 15% addition levels. From left to right: white wheat + powder, white wheat + pulp, colored wheat + powder, and colored wheat + pulp. All images are shown at the same scale.

Figure 3 provides representative cross-sectional images of the different formulations and was used to illustrate the general crumb appearance. The instrumental texture results were used as the primary basis for evaluating formulation-dependent textural differences. Water competition and changes in gluten-network continuity may contribute to the observed texture responses, as suggested by previous studies, but these mechanisms require further direct verification [2,9,10,28,29].

### 3.5. Anthocyanin Content and ABTS Radical Scavenging Activity

**Figure 4:**
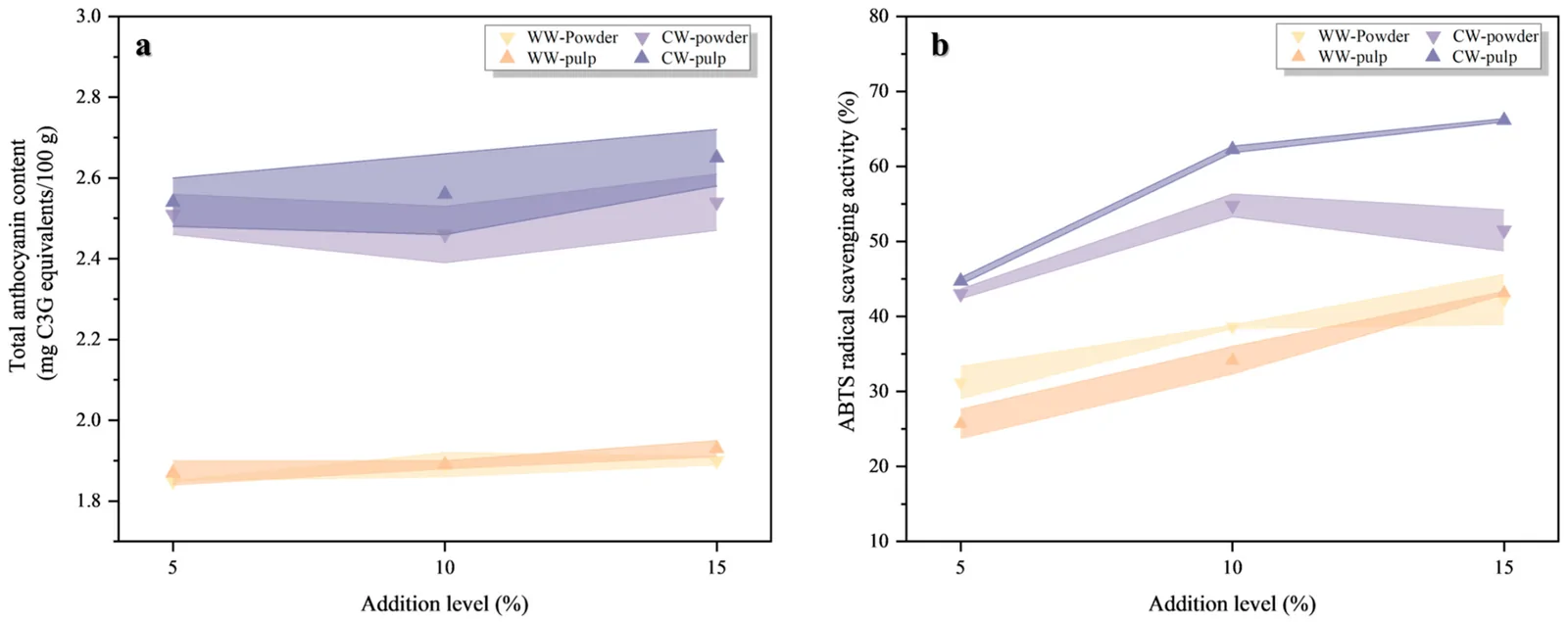
Changes in (**a**) total anthocyanin content and (**b**) ABTS radical scavenging activity of steamed-bread samples prepared from white and colored wheat with jujube powder or jujube pulp at different addition levels. Lines represent mean values, and shaded bands indicate ± SD (*n* = 3). WW, white wheat; CW, colored wheat.

The changes in anthocyanin content and ABTS radical scavenging activity are shown as mean values with corresponding SD in Figure 4. Anthocyanin content was clearly governed by wheat matrix. Across all addition levels, the colored-wheat samples remained much higher than the corresponding white-wheat samples, while the differences among 5%, 10%, and 15% within each series were relatively small. In the white-wheat system, anthocyanin content increased only slightly with increasing jujube level, regardless of whether jujube was added as powder or pulp. In the colored-wheat system, the same overall stability was observed, although the pulp-treated samples tended to be slightly higher than the corresponding powder-treated samples, especially at 15% addition. Although the dried jujube contained 6.39 mg/100 g anthocyanins, the jujube addition was only 5–15%, while colored whole-wheat flour remained the main component of the formulation. This may explain why anthocyanin content changed only slightly as the jujube level increased, while the difference between the two wheat matrices remained much larger. These results indicate that the anthocyanin content of the final product was determined mainly by the colored-wheat background, while jujube form had only a secondary influence. This is in line with recent studies showing that anthocyanin retention in wheat-based foods depends strongly on the original pigmented matrix and on processing-associated stability constraints [11,12,15].

In contrast, ABTS radical scavenging activity showed a clearer formulation response. In the white-wheat powder series, ABTS activity increased progressively from 31.19% at 5% addition to 42.28% at 15%. A similar increasing trend was observed in the white-wheat pulp series, where the value rose from 25.69% at 5% to 43.12% at 15%. In the colored-wheat powder series, antioxidant activity was consistently higher than that of the corresponding white-wheat series and increased from 43.01% at 5% to 54.81% at 10%, followed by a slight decline to 51.32% at 15%. The most pronounced enhancement was observed in the colored-wheat pulp series, in which ABTS activity increased continuously from 44.72% at 5% to 66.15% at 15%, giving the highest value among all treatments. These results indicate that both wheat matrix and jujube form affected antioxidant performance, and that jujube pulp was particularly effective in enhancing ABTS radical scavenging activity in the colored-wheat system.

Taken together, anthocyanin content and ABTS radical scavenging activity showed different factorial responses. Anthocyanin content was affected primarily by wheat type, whereas ABTS activity was significantly affected by wheat type, jujube form, addition level, and all two-way interactions. These interaction effects indicate that the antioxidant response to jujube incorporation depended on both the wheat matrix and addition level. A recent study on jujube powder-enriched steamed bread similarly reported addition-level-dependent changes in antioxidant activity and texture [20]. Anthocyanin content was positively correlated with ABTS radical scavenging activity (r = 0.82, *p* < 0.01), although the two responses did not change in parallel across treatments. This suggests that anthocyanins contributed to antioxidant activity together with other antioxidant-active constituents, including phenolic acids and flavonoids reported in jujube [17,18,19]. Accordingly, the ABTS response observed in this study should be considered an integrated antioxidant response of the wheat–jujube system rather than being attributed to anthocyanins alone.

### 3.6. Multivariate Analysis of Quality and Antioxidant-Related Responses

**Figure 5:**
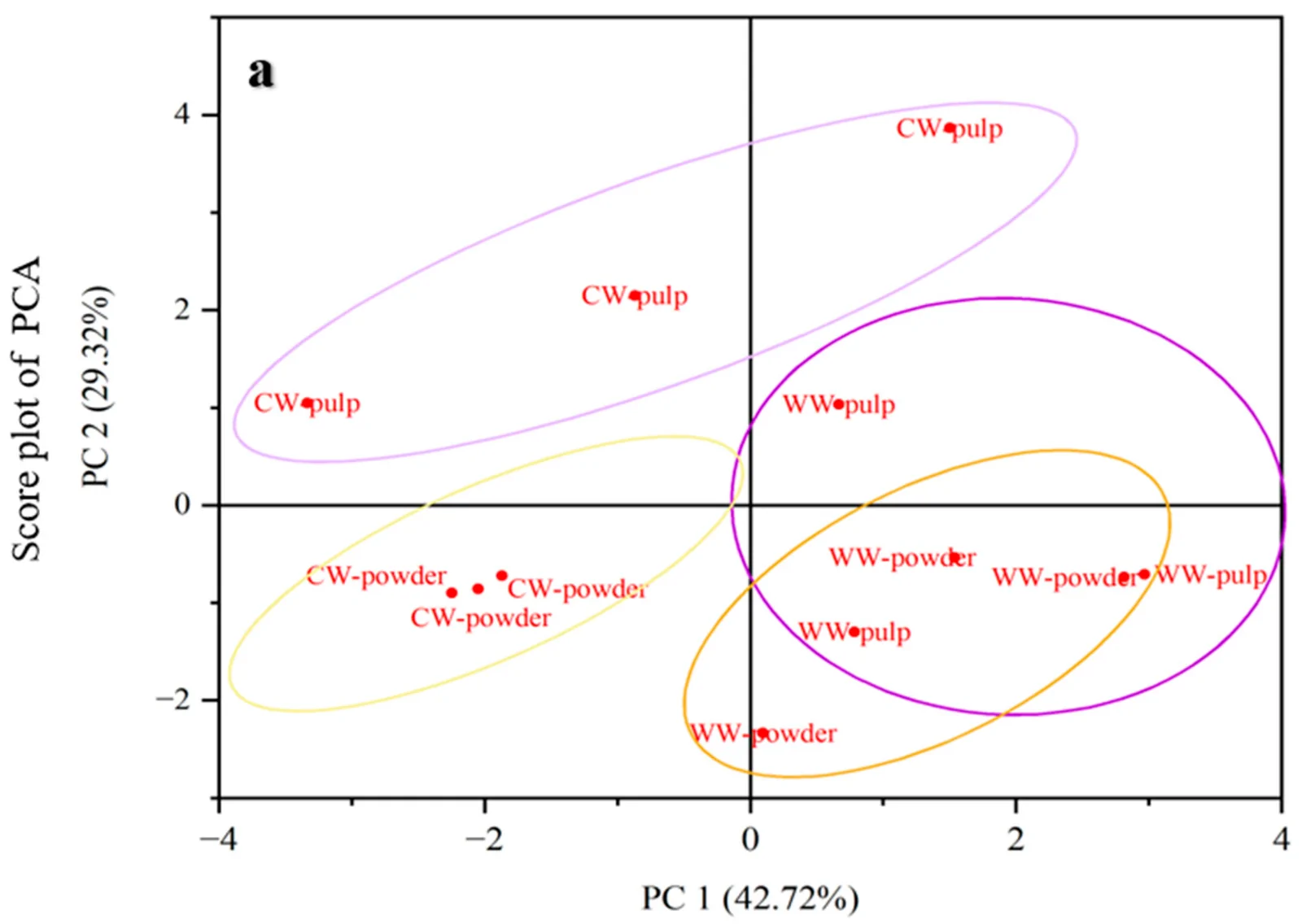

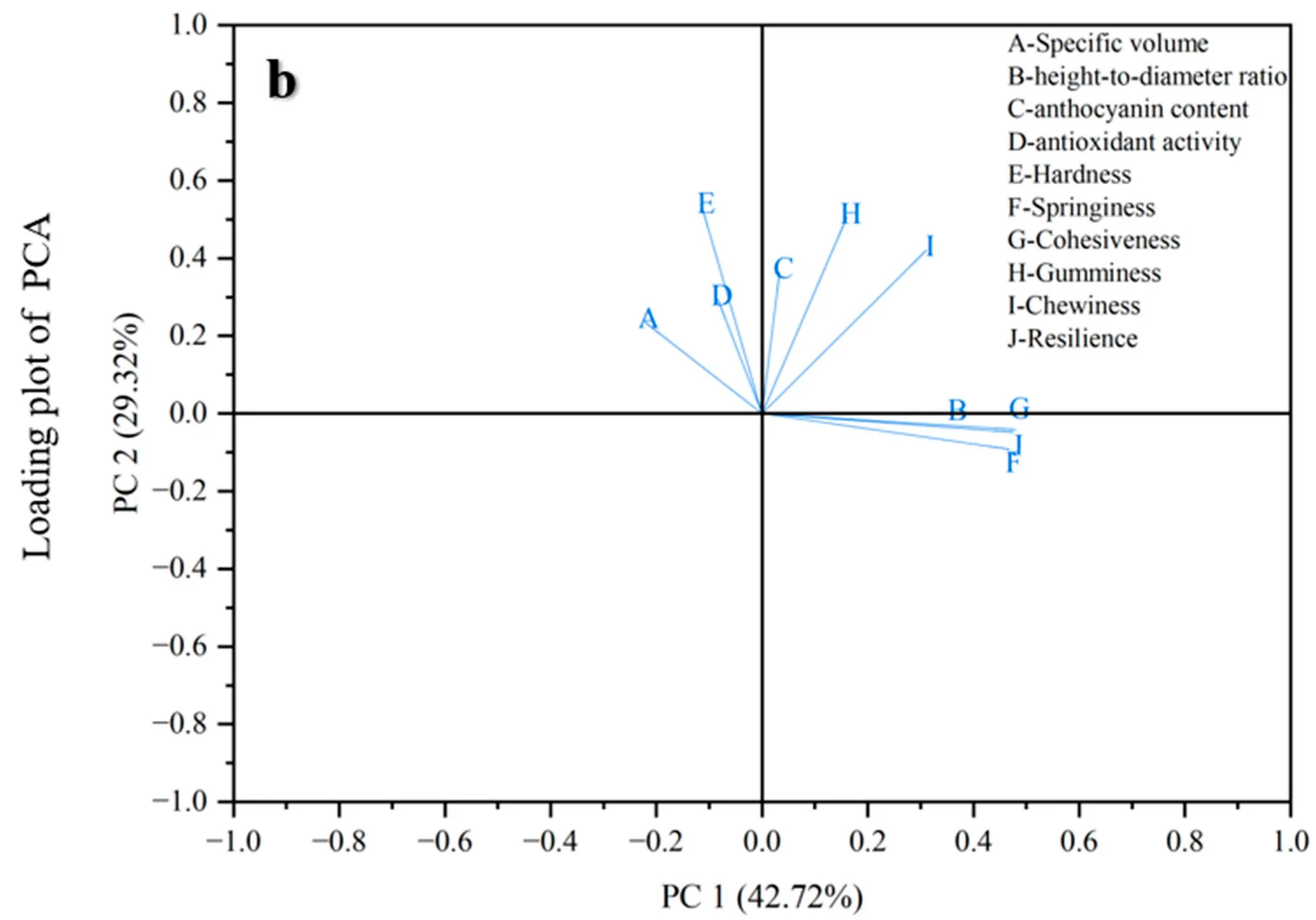
Score plot (**a**) and loading plot (**b**) of principal component analysis (PCA) based on physicochemical, textural and functional indicators of steamed-bread samples prepared from white wheat and colored wheat incorporated with jujube powder or jujube pulp. Ellipses in score plot were used only to aid visual grouping and should not be interpreted as statistical confidence intervals. WW, white wheat; CW, colored wheat. Labels A–J refer to specific volume, height-to-diameter ratio, anthocyanin content, ABTS, hardness, springiness, cohesiveness, gumminess, chewiness and resilience, respectively.

**Figure 6:**
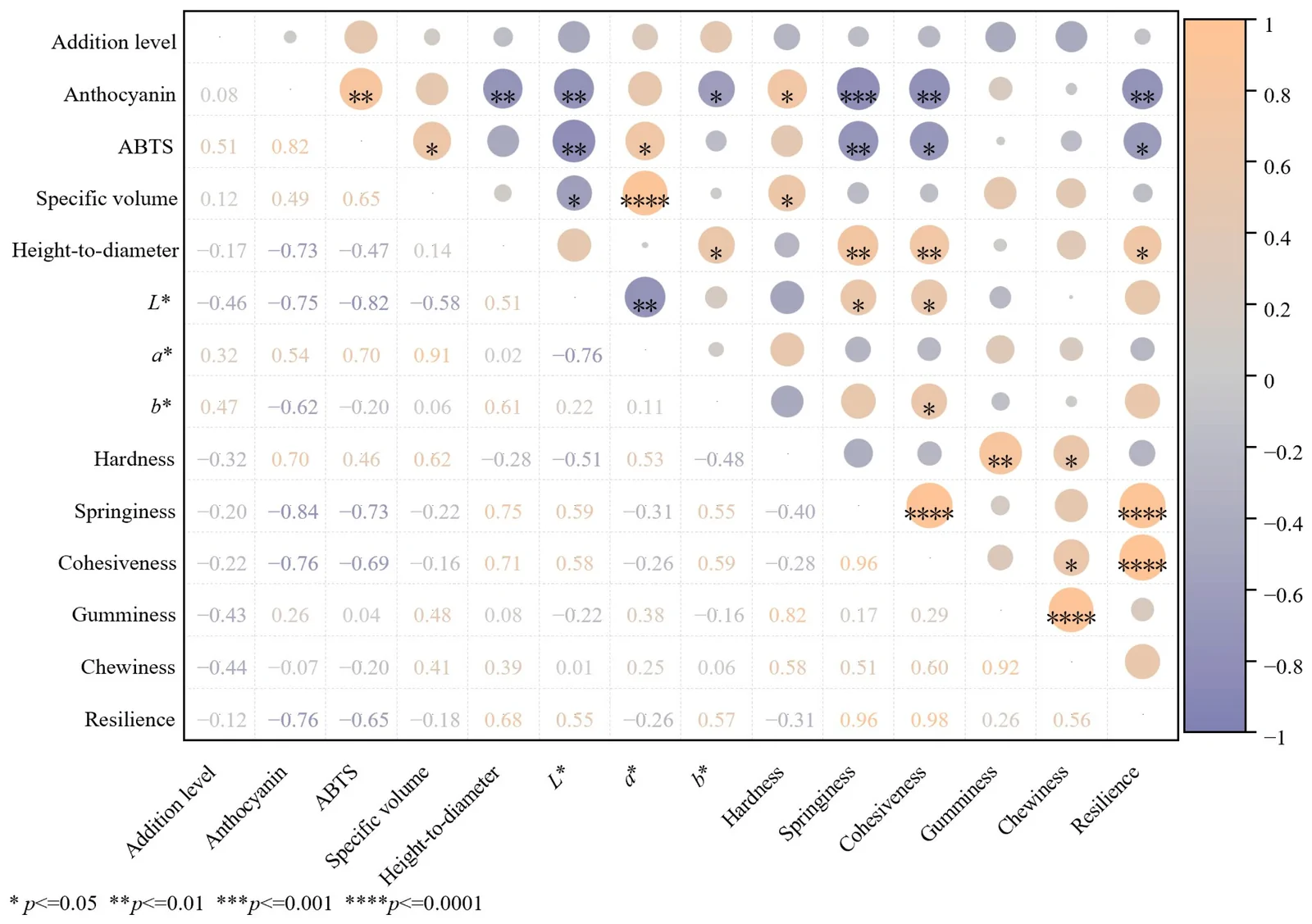
Pearson correlation heatmap of steamed bread quality and antioxidant-related parameters. Values in cells represent Pearson’s correlation coefficients. *, **, ***, and **** indicate *p* ≤ 0.05, *p* ≤ 0.01, *p* ≤ 0.001, and *p* ≤ 0.0001, respectively.

PCA was performed to further evaluate the overall quality-response pattern of different formulations (Figure 5). PC1 and PC2 explained 42.72% and 29.32% of the total variance, respectively, with a cumulative contribution of 72.04%. Thus, the first two components captured most of the variation among the measured variables. The score plot showed a clear tendency for samples to separate according to wheat matrix and jujube form, indicating that these two factors were important sources of overall quality variation. The loading plot showed that specific volume, height-to-diameter ratio, anthocyanin content, ABTS activity, and texture parameters contributed to sample discrimination. Hardness, gumminess, and chewiness showed similar loading directions, while springiness, cohesiveness, and resilience formed another related group, consistent with the correlations shown in Figure 6.

Pearson correlation analysis further clarified the relationships among quality and antioxidant-related parameters (Figure 6). Anthocyanin content was positively correlated with ABTS radical scavenging activity, indicating that the colored wheat matrix contributed to antioxidant-related properties. ABTS activity was negatively correlated with L* (r = −0.82, *p* < 0.01), showing that samples with higher antioxidant activity tended to have lower lightness. This association may mainly reflect the common influence of wheat matrix and jujube formulation on both color and antioxidant response. Strong positive correlations were also observed among springiness, cohesiveness, and resilience, while hardness was positively associated with gumminess, indicating that texture parameters were closely interrelated. Overall, the multivariate results showed that stronger antioxidant responses were generally accompanied by darker color and distinct texture profiles, indicating a trade-off among formulation targets.

## 4. Conclusions

Wheat type, jujube incorporation form, and addition level jointly affected the quality and antioxidant-related properties of whole-wheat steamed bread. Colored-wheat flour provided a markedly higher anthocyanin background and generally higher ABTS radical scavenging activity, but was also associated with darker color and distinct textural responses. Jujube incorporation form further modified these responses, and significant interaction effects demonstrated that the effects of powder and pulp were dependent on both wheat matrix and addition level. Within the colored-wheat pulp series, the 10% treatment showed the highest mean height-to-diameter ratio together with a substantial enhancement in ABTS activity, whereas the 15% treatment produced the highest measured ABTS activity but lower springiness, cohesiveness, and resilience. Therefore, no formulation was superior for every measured attribute; rather, the results identify different formulation options according to the desired product target. Under the tested processing conditions, 10% jujube pulp provided a more balanced response when both product shape and antioxidant activity were considered, whereas increasing pulp to 15% further enhanced antioxidant activity at the expense of several instrumental texture attributes. These findings should be interpreted within the tested 5–15% jujube addition range and the fixed processing conditions used in this study, while further sensory evaluation is needed to assess the acceptability of these formulations and support their practical application in colored whole-wheat steamed bread.

## Figures and Tables

**Table 1:** Proximate composition and wet gluten content of the two whole-wheat flours.

Sample	Starch (%)	Protein (%)	Dietary Fiber (%)	Fat (%)	Ash (%)	Wet Gluten (%)
White wheat	59.41 ± 0.04 ^a^	12.69 ± 0.08 ^b^	7.98 ± 0.23 ^a^	2.65 ± 0.07 ^b^	1.31 ± 0.05 ^b^	27.32 ± 0.01 ^b^
Colored wheat	59.08 ± 1.29 ^a^	13.97 ± 0.18 ^a^	6.42 ± 0.47 ^b^	2.90 ± 0.14 ^a^	1.42 ± 0.00 ^a^	33.95 ± 0.00 ^a^

Note: Values are mean ± SD (*n* = 3). Different lowercase letters within the same column indicate significant differences (*p* < 0.05).

**Table 2:** Mineral and anthocyanin contents of the two whole-wheat flours.

Sample	Fe (μg/g)	Zn (μg/g)	Se (μg/g)	Anthocyanins [mg/100 g]
White wheat	7.94 ± 0.18 ^a^	4.97 ± 0.22 ^a^	1.54 ± 0.05 ^b^	0.99 ± 0.00 ^b^
Colored wheat	7.05 ± 0.34 ^b^	3.98 ± 0.04 ^b^	4.21 ± 0.18 ^a^	4.24 ± 0.13 ^a^

Note: Values are mean ± SD (*n* = 3). Different lowercase letters within the same column indicate significant differences (*p* < 0.05).

**Table 3:** Three-way ANOVA results for the effects of wheat matrix, jujube form, addition level, and their interactions on quality and antioxidant-related parameters.

Variable	W *p* (*η_p_*^2^)	J *p* (*η_p_*^2^)	A *p* (*η_p_*^2^)	W × J *p* (*η_p_*^2^)	W × A *p* (*η_p_*^2^)	J × A *p* (*η_p_*^2^)	W × J × A *p* (*η_p_*^2^)
Height-to-diameter ratio	<0.001 (0.969)	<0.001 (0.667)	<0.001 (0.814)	<0.001 (0.938)	0.210 (0.229)	0.180 (0.249)	0.001 (0.676)
Specific volume	0.022 (0.265)	0.012 (0.425)	0.629 (0.074)	0.002 (0.559)	0.166 (0.259)	0.729 (0.051)	0.970 (0.005)
L*	<0.001 (0.946)	<0.001 (0.719)	<0.001 (0.885)	<0.001 (0.480)	0.004 (0.366)	<0.001 (0.766)	<0.001 (0.656)
a*	<0.001 (0.813)	<0.001 (0.825)	<0.001 (0.684)	<0.001 (0.897)	0.029 (0.256)	0.023 (0.268)	0.287 (0.099)
b*	<0.001 (0.666)	0.438 (0.025)	<0.001 (0.513)	0.001 (0.386)	0.081 (0.189)	0.169 (0.138)	0.025 (0.264)
Anthocyanin	<0.001 (0.987)	0.410 (0.303)	0.700 (0.358)	0.157 (0.160)	0.529 (0.101)	0.757 (0.047)	0.717 (0.054)
ABTS	<0.001 (0.983)	0.004 (0.519)	<0.001 (0.965)	<0.001 (0.844)	0.003 (0.617)	<0.001 (0.742)	0.160 (0.263)
Hardness	<0.001 (0.987)	<0.001 (0.943)	<0.001 (0.952)	<0.001 (0.900)	<0.001 (0.902)	<0.001 (0.776)	<0.001 (0.955)
Springiness	<0.001 (0.986)	0.140 (0.172)	<0.001 (0.836)	0.004 (0.519)	<0.001 (0.893)	<0.001 (0.899)	<0.001 (0.910)
Cohesiveness	<0.001 (0.995)	0.002 (0.578)	<0.001 (0.946)	<0.001 (0.843)	<0.001 (0.965)	<0.001 (0.933)	<0.001 (0.989)
Gumminess	<0.001 (0.984)	<0.001 (0.991)	<0.001 (0.994)	<0.001 (0.987)	<0.001 (0.991)	<0.001 (0.808)	<0.001 (0.997)
Chewiness	0.028 (0.342)	<0.001 (0.959)	<0.001 (0.975)	<0.001 (0.937)	<0.001 (0.963)	<0.001 (0.719)	<0.001 (0.983)
Resilience	<0.001 (0.996)	<0.001 (0.857)	<0.001 (0.863)	<0.001 (0.659)	<0.001 (0.969)	<0.001 (0.974)	<0.001 (0.990)

Note: W, wheat matrix; J, jujube form; A, addition level; W × J, wheat matrix × jujube form; W × A, wheat matrix × addition level; J × A, jujube form × addition level; W × J × A, wheat matrix × jujube form × addition level; *η_p_*^2^, partial eta squared.

**Table 4:** Texture profile analysis of jujube-enriched whole-wheat steamed bread.

Treatment	Hardness (g)	Springiness (mm)	Cohesiveness	Gumminess (g)	Chewiness (g·mm)	Resilience
WW-powder 5%	1494.25 ± 31.19 ^c^	0.86 ± 0.02 ^b^	0.75 ± 0.01 ^b^	1092.98 ± 3.10 ^a^	899.59 ± 43.01 ^a^	0.34 ± 0.00 ^b^
WW-powder 10%	1303.25 ± 34.46 ^d^	0.97 ± 0.04 ^a^	0.78 ± 0.01 ^a^	1019.27 ± 15.49 ^b^	979.09 ± 70.75 ^a^	0.42 ± 0.02 ^a^
WW-powder 15%	950.98 ± 0.76 ^e^	0.86 ± 0.01 ^b^	0.68 ± 0.01 ^c^	642.65 ± 6.25 ^e^	547.19 ± 5.19 ^d^	0.30 ± 0.01 ^c^
CW-powder 5%	1654.66 ± 5.77 ^a^	0.68 ± 0.00 ^c^	0.59 ± 0.00 ^d^	911.40 ± 14.46 ^c^	648.71 ± 7.59 ^bc^	0.21 ± 0.00 ^e^
CW-powder 10%	1576.14 ± 60.64 ^b^	0.72 ± 0.01 ^c^	0.59 ± 0.00 ^d^	925.32 ± 2.44 ^c^	668.02 ± 3.71 ^b^	0.23 ± 0.00 ^d^
CW-powder 15%	1469.67 ± 0.38 ^c^	0.68 ± 0.01 ^c^	0.59 ± 0.00 ^d^	878.11 ± 2.18 ^d^	574.43 ± 19.54 ^cd^	0.23 ± 0.00 ^d^
WW-pulp 5%	1052.19 ± 57.07 ^e^	0.88 ± 0.00 ^b^	0.70 ± 0.00 ^c^	747.28 ± 24.75 ^e^	669.50 ± 35.19 ^c^	0.33 ± 0.00 ^c^
WW-pulp 10%	1614.62 ± 8.96 ^c^	0.84 ± 0.01 ^c^	0.68 ± 0.00 ^d^	1101.03 ± 8.18 ^b^	942.47 ± 5.72 ^b^	0.32 ± 0.00 ^d^
WW-pulp 15%	1258.81 ± 28.38 ^d^	0.93 ± 0.01 ^a^	0.80 ± 0.01 ^a^	1000.40 ± 11.21 ^c^	927.85 ± 1.17 ^b^	0.44 ± 0.00 ^a^
CW-pulp 5%	2259.16 ± 60.57 ^a^	0.85 ± 0.00 ^c^	0.73 ± 0.01 ^b^	1696.43 ± 19.67 ^a^	1426.20 ± 18.54 ^a^	0.34 ± 0.00 ^d^
CW-pulp 10%	1893.10 ± 65.65 ^b^	0.73 ± 0.00 ^d^	0.62 ± 0.00 ^e^	1112.08 ± 6.06 ^b^	959.48 ± 3.06 ^b^	0.23 ± 0.00 ^e^
CW-pulp 15%	1713.80 ± 5.16 ^c^	0.59 ± 0.01 ^e^	0.51 ± 0.00 ^f^	882.25 ± 0.66 ^d^	520.77 ± 9.95 ^d^	0.17 ± 0.00 ^f^

Note: Values are mean ± SD (*n* = 6). Within each jujube-form series (powder or pulp), values in the same column followed by different lowercase letters are significantly different (*p* < 0.05). WW, white wheat; CW, colored wheat.

## Data Availability

The original contributions presented in this study are included in the article. Further inquiries can be directed to the corresponding author.
